# Supplementary material for: From sequence to enzyme mechanism using multi-label machine learning
Source: BMC Bioinformatics. 2014 May 19;15:150. doi: 10.1186/1471-2105-15-150 (PMC4229970; doi:10.1186/1471-2105-15-150)
Supplement: Additional file 2 — Java code of ml2db. Additional file ml2db_code.tar.gz contains the Java source code to run the multi-label machine learning experiments and save the results to database. The code’s Javadoc is included. [file 1471-2105-15-150-S2.zip › additional file 2/ml2db/ml2db/doc/mulan/data/class-use/LabelNode.html]

Uses of Interface mulan.data.LabelNode


JavaScript is disabled on your browser.


- Overview
- Package
- Class
- Use
- Tree
- Deprecated
- Index
- Help

- Prev
- Next

- Frames
- No Frames

- All Classes

## Uses of Interface mulan.data.LabelNode

- Packages that use LabelNode

  | Package | Description |
  | --- | --- |
  |  |  |
  | --- | --- |
  | mulan.data |  |
- - ### Uses of LabelNode in mulan.data

    Classes in mulan.data that implement LabelNode

    | Modifier and Type | Class and Description |
    | --- | --- |
    |  |  |
    | --- | --- |
    | `class` | `LabelNodeImpl` Implementation of `LabelNode`, representing a label attribute and its connection within a hierarchy of labels. |

    Methods in mulan.data that return LabelNode

    | Modifier and Type | Method and Description |
    | --- | --- |
    |  |  |
    | --- | --- |
    | `LabelNode` | LabelsMetaDataImpl.`getLabelNode(java.lang.String labelName)` |
    | `LabelNode` | LabelsMetaData.`getLabelNode(java.lang.String labelName)` Gets the `LabelNode` specified by label name. |
    | `LabelNode` | LabelNodeImpl.`getParent()` |
    | `LabelNode` | LabelNode.`getParent()` Gets the parent `LabelNode` of this node if hierarchy exists. |

    Methods in mulan.data that return types with arguments of type LabelNode

    | Modifier and Type | Method and Description |
    | --- | --- |
    |  |  |
    | --- | --- |
    | `java.util.Set<LabelNode>` | LabelNodeImpl.`getChildren()` |
    | `java.util.Set<LabelNode>` | LabelNode.`getChildren()` Gets the unmodifiable `Set` of child `LabelNode` of this node, if hierarchy exists. |
    | `java.util.Set<LabelNode>` | LabelsMetaDataImpl.`getRootLabels()` |
    | `java.util.Set<LabelNode>` | LabelsMetaData.`getRootLabels()` Gets the unmodifiable `Set` of root `LabelNode` nodes of label attributes hierarchy. |

    Methods in mulan.data with parameters of type LabelNode

    | Modifier and Type | Method and Description |
    | --- | --- |
    |  |  |
    | --- | --- |
    | `boolean` | LabelNodeImpl.`addChildNode(LabelNode node)` Adds the specified `LabelNode` to the set of child nodes. |
    | `void` | LabelsMetaDataImpl.`addRootNode(LabelNode rootNode)` Adds a root `LabelNode`. |
    | `boolean` | LabelNodeImpl.`removeChildNode(LabelNode node)` Removes the specified `LabelNode` from the set of child nodes. |

- Overview
- Package
- Class
- Use
- Tree
- Deprecated
- Index
- Help

- Prev
- Next

- Frames
- No Frames

- All Classes
